# Murine Dendritic Cells Grown in Serum-Free Culture Show Potent Therapeutic Activity when Loaded with Novel Th Epitopes in an Orthotopic Model of HER2^pos^ Breast Cancer

**DOI:** 10.3390/vaccines9091037

**Published:** 2021-09-18

**Authors:** Loral E. Showalter, Brian J. Czerniecki, Krithika Kodumudi, Gary K. Koski

**Affiliations:** 1 Department of Biological Sciences, School of Biomedical Sciences, Kent State University, Kent, OH 44242, USA; lshowal1@kent.edu; 2 Department of Breast Oncology, H. Lee Moffitt Cancer Center, 12902 Magnolia Drive, Tampa, FL 33612, USA; Brian.Czerniecki@moffitt.org; 3 Clinical Science Lab Department, H. Lee Moffitt Cancer Center, 12902 Magnolia Drive, Tampa, FL 33612, USA; krithika.kodumudi@moffitt.org

**Keywords:** dendritic cell, vaccine, peptide, epitope, breast cancer, ErbB2/HER2

## Abstract

Preferred methods for generating mouse dendritic cells (DC) would encompass qualities of consistency, high yield, and potent function. Serum-free culture is also highly desirable, since this is the standard for cell-based therapies used in humans. We report here a serum-free modification of a culture method generating mature, activated DCs from bone marrow precursors. This is achieved through a two-stage culture comprised of 6-day expansion in Flt3 ligand and IL-6 followed by brief differentiation in a medium containing GM-CSF and IL-4, with subsequent activation using TLR ligands ODN1826 and LPS. The serum-free DCs achieve yields and surface phenotype including IL-12p70 secretion similar to standard serum-replete cultures, display a capacity to sensitize in vivo against both MHC class I- and Class II-restricted antigens, and exhibit some aspects of “killer DC” function against tumor cells. We used these DCs to help identify novel CD4^pos^ Th epitopes on the rat ErbB2/HER-2 protein and demonstrated a subset of these as effective immunogens in a DC-based therapeutic model of HER-2^pos^ breast cancer in Balb/c mice, where they induced powerful Th1-polarized immune responses. This method represents a useful way to efficiently produce large numbers of murine dendritic cells with excellent in vivo function well-suited for use in experimental vaccine studies.

## 1. Introduction

A promising strategy for cancer immunotherapy is the use of dendritic cells (DC), since these are known to be highly efficient for priming T lymphocytes to antigen [1]. From the standpoint of therapy, human DCs are most readily obtained from CD14^pos^ peripheral blood monocytes. This is because these cells are abundant in circulation and can be easily and safely obtained in numbers sufficient for laboratory or clinical studies. Typically, a multi-day culture (usually 6–7 days) transforms these monocytes into cells with the properties of immature DCs and is usually achieved in culture medium supplemented with the cytokines Granulocyte-macrophage colony stimulating factor (GM-CSF) and Interleukin-4 (IL-4) [2]. Nonetheless, modified methods with more rapid timeframes have been reported [3,4,5]. Full DC maturation and activation is typically achieved by addition of such agents as pro-inflammatory cytokines, or combinations of Toll-like receptor ligands (TLRs) [2,6,7]. It is also both necessary and convenient to have simple methodologies to produce functionally comparable cells from mice, either for the purpose of studying DC biology, or to model experimental DC-based immunotherapies that will form the foundation for future clinical studies in humans.

In the case of mice, blood is a rather inefficient choice for a source of DC. This is largely due to low yields, owing to the limited volumes of blood that can be obtained from individual animals. Methods have been reported to obtain murine DCs from splenic populations [8], but the most commonly used techniques employ precursor cells obtained from bone marrow (BM). Here, unfractionated populations from BM aspirates are cultured for several days in cytokines including GM-CSF, sometimes with added IL-4 [9,10,11]. The resulting cells can be matured and activated with many of the same stimuli that promote such changes in human DCs.

Alternate methods have also been devised to obtain large numbers of cells with the properties of DCs from mouse bone marrow. For example, Cohen et al. reported that 6-day culture of BM cells with a combination of fms-like tyrosine kinase 3 (Flt3) ligand and Interleukin-6 (IL-6) promoted a rapid and vigorous expansion of precursor cells resident in the bone marrow. If these cells are harvested and transferred to a second-stage culture containing GM-CSF and IL-4, the cells adopt, within in a day or two, the properties of immature DCs. These could be further activated with combinations of TLR ligands with subsequent high-level secretion of Interleukin-12 (IL-12) [12].

Whether human or murine, all of the original methods developed for culturing DCs were done using a serum-containing medium (typically either from human or fetal calf sources). However, there are several reasons why it would be desirable to eliminate serum as a component of DC culture medium. First, from the standpoint of human therapy, a medium free from animal or human blood components would represent a safer composition, lessening the possibility of transmitting infection or inducing allergic responses [13]. Second, a more defined medium would promise greater consistency. Finally, xenoantigenic serum components would naturally be taken up and processed by DC. When these were introduced to a recipient as a vaccine (human or animal), the DCs would not only sensitize T cells to whichever antigens were purposely loaded onto them, but also to the xenoantigenic serum components. This could induce spurious immune responses that would potentially either alter or interfere with the generation of desired responses (including therapeutic ones) or confound efforts to accurately assess the quality of immune response against the intended antigens. These latter complications have been reported by a number of investigators [14,15,16,17,18]. Serum free (SF) culture methods have been developed for culturing human DCs for therapy [4,19,20], and these and similar SF methods represent the current standard. In order to complement these, reliable SF methods to cultivate murine DCs that have potent function in vivo are highly desirable.

In the present studies we describe an SF modification of the two-stage culture system originally reported by Cohen et al. [12]. Here, we demonstrate that stage I culture (Flt3/IL-6) can be accomplished in a variety of commercial SF media, with stage II culture (GM-CSF/IL-4) performed in either an identical commercial SF medium or even base RPMI without added serum. These DCs are functional in vivo and sensitize to known major histocompatibility (MHC) class I and II antigens and were used to identify novel helper T cell epitopes on the rat ErbB2/HER2 oncodriver. When these newly-identified Th epitopes were used on a DC-based vaccine, a subset displayed considerable therapeutic activity in a murine model of HER2^pos^ breast cancer.

## 2. Materials and Methods

### 2.1. Mice

All mice used in experiments were female Balb/c mice between the ages of 14–16 weeks at the start of experiment (Charles River, Wilmington, MA, USA). They were maintained in accordance with the National Institute of Health’s guidelines, in a specific pathogen-free environment in the animal facility in Cunningham Hall at Kent State University. Experiments were approved by the Institutional Animal Care and Use Committee (IACUC) of Kent State University under the protocol 299GK 10–22 and 451GK-17-15.

### 2.2. Culture Media

Seven different media were used, including RPMI base with 2 mM glutamine, 0.1 mM nonessential amino acids, 100 units/mL sodium pyruvate, and 100 mg/mL PenStrep (all from Corning, Manassas, VA, USA) with either no fetal bovine serum (FCS), or 10% FCS (VWR/Seradigm, Atlanta, GA, USA) added; RPMI base with HL-1 FCS substitute (Lonza, Walkersville, MD, USA), as well as several commercial serum-free media including Macrophage-SFM (Gibco Invitrogen, Grand Island, NE, USA); X-Vivo 10; X-Vivo 15, or X-Vivo 20 (all from Lonza).

### 2.3. Cell Lines

ErbB2/HER2^pos^ TUBO breast carcinoma cell line was a gift from Dr. Wei Zen Wei, (Wayne State University) and maintained by serial passage in RPMI complete medium with 10% FCS.

### 2.4. DC Culture Methods

A two-stage culture strategy was employed for production of dendritic cells from BM [12]. Briefly, BM cells were harvested from the femurs and tibia of Balb/c mice and cultured at 5 × 10^5^ cells/mL for 6 days in one of the seven culture media supplemented with 30 ng/mL human Flt-3L and 25 ng/mL murine IL-6 (both from Peprotech, Rocky Hill, NJ, USA) (Stage I). On day 6 cells were harvested, washed two times in phosphate buffered saline (PBS), resuspended in RPMI (without FCS) supplemented with 50 ng/mL murine GM-CSF and 10 ng/mL IL-4 (both from Peprotech), either plated in 12-well dishes for analytic studies or placed in culture flasks for vaccine production, and incubated overnight at 2 × 10^6^ cells/mL (Stage II). The next day, cells were activated by the addition of E. coli K12 lipopolysaccharide (LPS) (20 ng/mL) and CpG motif-containing oligonucleotide ODN1826 (10 ng/mL) (each from In VivoGen, San Diego, CA, USA).

### 2.5. Synthetic Peptides and Identification of Immunogenic Peptides

A peptide library was created representing the extracellular domain of rat ErbB2/HER2 (21st Century Biochemicals, Marlborough, MA, USA). It consisted of 62 peptides, each 20 amino acids in length, with consecutive peptides overlapping by 10 amino acids (Supplementary Materials Figure S3). Rat ErbB2/HER2 cytotoxic T cell (CTL) epitope (TYVPANASL) was synthesized by Bachem (Torrance, CA, USA) and ovalbumin helper T (Th) epitope ISQAVHAAHAEINEAGR was purchased from InvivoGen (San Diego, CA, USA). Individual peptides were dissolved at 10 mg/mL in DMSO and stored at −70 °C until use. From the library, 12 pools were created containing 5–6 consecutive peptides. These 12 pools were used to vaccinate 60 Balb/c mice (five mice per group). Mice (five per group) received two immunizations (two weeks apart) with peptide pools emulsified in Freund’s Adjuvant (Complete for the first and Incomplete for the second) (MP Biomedicals, Solon, OH, USA). Each dose contained 20 µg per peptide in a total volume of 100 µL per mouse. Sera were obtained every two weeks via submandibular blood collection followed by centrifugation, and resulting serum was analyzed via ELISA for anti-peptide immunoglobin G (IgG).

### 2.6. ELISA for Antibody Detection (Peptide Library)

Vaccinating as well as negative control peptides were individually coated onto Costar Assay 96-well plates (Corning, Corning, NY, USA) at 10 µg/mL in bicarbonate buffer and incubated overnight a 4 °C. The following day plates were blocked with 10% FCS in PBS. Sera from vaccinated mice (diluted 1:100) was added in triplicate to wells and incubated for 2 h at room temperature (RT). The plates were then washed three times before the addition of 1:500 diluted horse radish peroxidase (HRP)-conjugated anti-mouse secondary antibody (Santa Cruz Biotechnology, Dallas, TX, USA). After incubation for 1 h, plates were washed five times and developed with 3,3′,5,5′-Tetramethylbenzidine (TMB) substrate solution (Kirkegaard & Perry Laboratories, Gaithersburg, MD,USA). The assay was stopped with 25 µL of 1N hydrochloric acid solution and read at 450 nm on a Biotek ELx800 microplate reader running Gen5 software.

### 2.7. Alamar Blue Assay to Determine Tumor Viability in Response to Secreted DC Products

TUBO cells were plated in a 96-well cluster plate at a density of 5 × 10^3^ cells per well in 50 µL of complete RPMI media with either 50 µL of additional media (No Rx), or 50 µL of 24 h culture supernatants from DCs that were either not activated or activated with LPS and ODN1826. TUBO cells were allowed to incubate for 72 h before the addition of 20 µL of 0.15 mg/mL of resazurin salt in PBS. After an additional 5 h of incubation the optical density of the culture supernatants was measured at 630 nm using a Biotek ELx800 microplate reader.

### 2.8. Photomicroscopy

Images were taken of dendritic cells in culture after 4 h of activation (LPS and ODN1826) using a Zeiss Primovert microscope (20× objective), an Axiocam 105 color camera, and Zen Blue imaging software.

### 2.9. Flow Cytometry

Harvested cells were washed in PBS and resuspended in PBS containing 1% FCS and 1% sodium azide. The cells were then stained with Fluorescein isothiocyanate (FITC)-conjugated anti-mouse I-A^d^, or CD11b, or phycoerythrin (PE)-conjugated anti-mouse CD40 (all Biolegend), or PE conjugated anti-mouse CD80 or CD86 (BD Biosciences), or corresponding isotype controls. After 30 min of incubation, cells were washed and fluorescence was measured via Amnis FlowSight Flow Cytometer (Luminex, Austin, TX, USA) and analyzed using IDEAS software package.

### 2.10. Detection of Secreted Cytokines

For dendritic cells, stage II cultures were stimulated with LPS and ODN1826, and culture supernatants harvested 24 h later. Supernatants were then analyzed either for IL-12p70 via Enzyme-linked immunosorbent assay (ELISA) (OptEIA BD Biosciences, San Diego, CA, USA) or for a set of 97 pro-inflammatory cytokines using mouse antibody cytokine array C6 (RayBiotech, Norcross, GA, USA). Both types of analysis were performed according to manufacturer’s recommendations.

For T cells, splenocytes from immune and control mice were cultured in RPMI base medium and stimulated with recall and control peptides (20 µg/mL) or left unstimulated, and 24 h culture supernatants were harvested and analyzed by ELISA for Interferon-gamma (IFN-γ), IL-2, IL-4 or IL-10 (OptEIA, BD Biosciences, San Diego, CA, USA) according to manufacturer’s recommendations.

The optical density of the ELISA assay was measured via a Bio-Tek ELx800 absorbance reader. The Cytokine Array membrane was visualized using an ImageQuant LAS 4000 mini (GE Healthcare).

### 2.11. Enumeration of Cytokine-Secreting Cells (Elispot)

As previously described [21], immune mice were sacrificed and single cell suspensions of spleens were made for use in a murine IFN-γ T cell single-color enzymatic ImmunoSpot assay (C.T.L., Cleveland, OH, USA). The assay was carried out via manufacturer’s instructions using 20 µg/mL of stimulating peptide and 400,000 splenocytes per well. In some experiments, manufacturer’s kit supplied medium was substituted with base RPMI (no added FCS). The plate was read on an ImmunoSpot analyzer (C.T.L.).

### 2.12. Implantation of Tumors

Tumors were implanted orthotopically as previously described [22]. Briefly, cultured TUBO breast carcinoma cells [23] were introduced into Balb/c mice in the region of the fat pad of the breast (2.5 × 10^5^ TUBO cells in 100 µL of PBS per mouse). Mice were then treated when the tumors were palpable (typically between 7–10 days post-implantation). Tumors were measured twice weekly using calipers to measure length (longest axis) and width (perpendicular), the product of which is taken as tumor area in mm^2^.

### 2.13. Preparation and Administration of Vaccines

DCs used for vaccination completed Stage I culture and entered overnight Stage II culture with GM-CSF and IL-4 in either RPMI supplemented with 10% FCS or RPMI base medium (no FCS). The next morning, synthetic peptides including ovalbumin (OVA) [^323^ISQAVHAAHAEINEAGR^339^], rat ErbB2/HER2 (rErbB2/HER2) Class I peptide [^66^TYVPANASL^74^] [24] (“p66”), rErbB2/HER2 peptides p9 [^80^QEVQGYMLIAHNQVKRVPLQR^100^], p17 [^160^GNPQLCYQDMVLWKDVFRKN^180^], p35 [^340^PCARVCYGLGMEHLRGARAI^360^], or p48 [^470^LIHRNAHLCFVHTVPWDQLF^490^]) were added to the culture (20 µg/mL). After 2 h incubation the DCs were activated with a combination of LPS (20 ng/mL) and CpG oligonucleotide ODN1826 (10 ng/mL) and incubated an additional 3 h, then harvested, washed 3× with PBS, and resuspended at 1 × 10^7^ cells/mL in PBS for vaccination using 0.5 cc syringes fitted with 27 ga needles. Each mouse received 100 µL of DCs injected subcutaneously in the flank. In some experiments, where indicated, DCs were injected directly into the tumor bed.

### 2.14. Winn Assay

Splenocytes from immune or control mice were mixed with TUBO cells at a ratio of 10:1, respectively, and a total of 5 × 10^5^ cells were implanted subcutaneously into the abdomens of naïve mice. Mice were observed periodically for tumor formation and nodules measured via caliper every 3–4 days.

### 2.15. Depletion of CD4^pos^ and CD8^pos^ Populations from Splenocytes

Single cell suspensions of splenocytes were prepared from the excised spleens of mice vaccinated once with DCs pulsed with P9 or P17 (three mice per peptide for a total of six mice). Spleen cell preparations from each individual mouse were divided in half for either CD4^pos^ depletion or CD8^pos^ depletion. The MidiMACS system (MidiMACS separator, LS Columns, CD8 (TIL) Microbeads, and CD4 (L3T4) Microbeads) was used for depletion via manufacturer’s instructions (Miltenyi Biotec, Bergisch Gladbach, Germany). Following separation, the preparations were analyzed via flow cytometry to ensure successful depletion and used in an in vitro recall assay for the vaccinating peptide (P9 or P17). Recall of antigen was assessed from 24 h stimulated culture supernatants using BD OptEIA mouse IFNγ ELISA set (BD Biosciences, San Diego, CA, USA) via manufacturer’s instruction. A stimulation index (IFN-γ output in stimulated divided by unstimulated cells) was determined for each individual mouse and then averaged.

### 2.16. Statistical Analysis

As previously described [21], One-way Analysis of Variance (ANOVA) was performed to determine if there were statistical differences between means, and a Holm–Sidak test was used priori to compare the difference between individual groups. Sigma Plot software was used to run all statistical analyses. Treatment groups with a *p* value of less than 0.05 were considered to be significantly different.

## 3. Results

### 3.1. Serum-Free Dendritic Cell Culture Achieves Yields and Phenotype Comparable to Serum-Replete Culture

For the generation of murine DCs, we cultured mouse BM cells for 6 days in a medium supplemented with Flt3 ligand and IL-6. This “stage I” culture promotes an expansion of progenitor cells while predisposing them toward DC differentiation [12]. Cells were then removed to fresh media supplemented instead with GM-CSF and IL-4 (stage II). Within a day or two the cells acquire potentiated expression of MHC and co-stimulatory molecules characteristic of myeloid-derived dendritic cells. Activation with TLR ligands completed their maturation with subsequent high secretion of IL-12p70. We began our studies by comparing five commercial serum-free media (SFM) with the “Standard” culture conditions which included RPMI medium supplemented with 10% fetal calf serum (FCS-DC). As a further comparison we also examined whether base RPMI medium without any added FCS would support stage I expansion.

**Figure 1:**
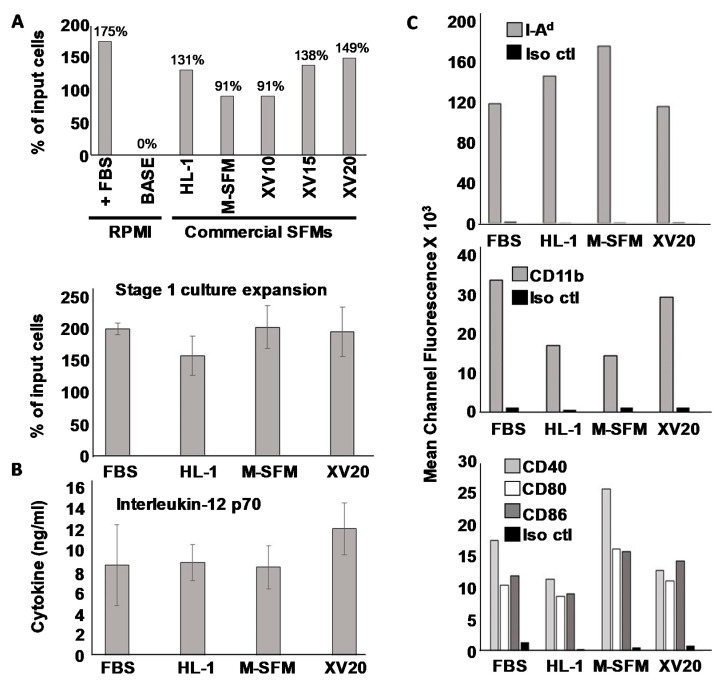
Characterization of DC phenotype. (**A**) One-time screen of seven different culture conditions for stage I DC culture with cells from murine bone marrow aspirates maintained for 6 days in indicated media supplemented with Flt/3 ligand and IL-6, then harvested and viable cell numbers determined using Trypan Blue dye exclusion. Percent expansion based on initial input cells was calculated (upper panel). Statistical analysis of stage I expansion of bone marrow cells from three separate experiments using four selected culture media (lower panel. (**B**) ELISA analysis for IL-12p70 of culture supernatants after stage II culture in GM-CSF and IL-4 followed by activation with ODN-1826 and LPS (**C**) Flow cytometry analysis of surface phenotype of cells 24 h after activation.

As our initial comparison of efficiency, after 6 days of stage I culture we harvested cells and assessed viability by Trypan Blue dye exclusion, and calculated overall percent expansion by comparing the total number of input cells with viable harvested cells at the end of the expansion period. We found that of the seven different conditions, FCS-containing RPMI medium appeared to perform marginally the best with 175% expansion. However, the highest performing serum-free media was not far behind, with X-Vivo 20 posting 149% expansion, X-Vivo 15 138%, HL-1 131%, and both X-Vivo 10 and macrophage SFM media at 91% (Figure 1A, upper panel). Only RPMI base medium without any FCS was completely unable to support measurable Stage I expansion or viability of the bone marrow cells across the 6-day culture period. Microscopically, the cells grown in commercial serum-free media all appeared similar to those grown in conventional serum-containing RPMI after subsequent stage II culture and activation (Supplementary Materials Figure S1).

After this initial screen, we chose three commercial SFMs for closer analysis. First, we re-tested stage I expansion in three replicate trials to determine whether any of the apparent difference observed in the initial single-trial screen would prove statistically significant. These replicate trials revealed that no statistically significant differences were observed between any SFM or between SFMs and FCS-containing RPMI (Figure 1A lower panel), indicating that all tested commercial SFMs were generally on par with serum-supplemented RPMI for supporting stage I expansion.

We then made observations after Stage II culture in GM-CSF and IL-4 followed by activation with the combination of LPS and ODN1826. Interestingly, we found that we could successfully carry out Stage II culture in RPMI base medium without any added FCS, and because we wished to limit, to the greatest degree possible, the introduction of xenoantigens, we used this base medium for stage II culture for all subsequent studies. After overnight incubation in stage II, LPS and ODN1826 were added to complete maturation and activation, and cells and supernatants were harvested 3–4 h later for analysis. Culture supernatants were evaluated for IL-12 p70 (Figure 1B), and DCs analyzed for surface marker expression via FACS through mean channel fluorescence (Figure 1C) with results also displayed as histograms (Supplementary Materials Figure S2). As with overall expansion efficiency, the capacity to produce IL-12 did not differ significantly between standard serum-containing conditions and any of the tested SFMs, although there did appear to be a modest trend for superior IL-12 secretion with the DCs grown in X Vivo 20 during Stage I compared with all other conditions. The picture for surface molecule expression was more complex. Whereas we did observe apparent differences in expression of various surface markers associated with mature DCs (e.g., I-A, CD11b and costimulatory molecules CD40, CD80 and CD86) depending on the culture medium, all culture conditions resulted in generally high expression of these molecules, and no one treatment (serum-containing or serum-free) held a clear advantage for the entire set of examined DC surface markers. We thus concluded that all of the serum-free media tested were roughly comparable to serum-supplemented RPMI.

Because our studies thus far could not identify a clearly superior serum-free culture medium for Stage I, it was difficult to rationally choose a single one to focus on for the remainder of this study. Nonetheless, since our own interests are in DCs that are maximally suitable for experimental cancer immunotherapy, and because we believe Th1-dominated immune responses are superior for fostering anti-tumor immunity, we selected the culture medium that trended for the highest induction of IL-12, the cytokine primarily responsible for driving polarized T helper 1 (Th1) differentiation [25]. X-vivo 20 medium was therefore chosen for DC culture in all subsequent studies, and henceforth, DCs grown in this SFM will be referred to as “SFM-DC” to distinguish them from those grown in RPMI medium supplemented with fetal calf serum (“FCS-DC”).

### 3.2. Dendritic Cells Cultured in Serum-Free Medium Sensitize against Either MHC Class I or MHC Class II-Restricted Antigens In Vivo

**Figure 2:**
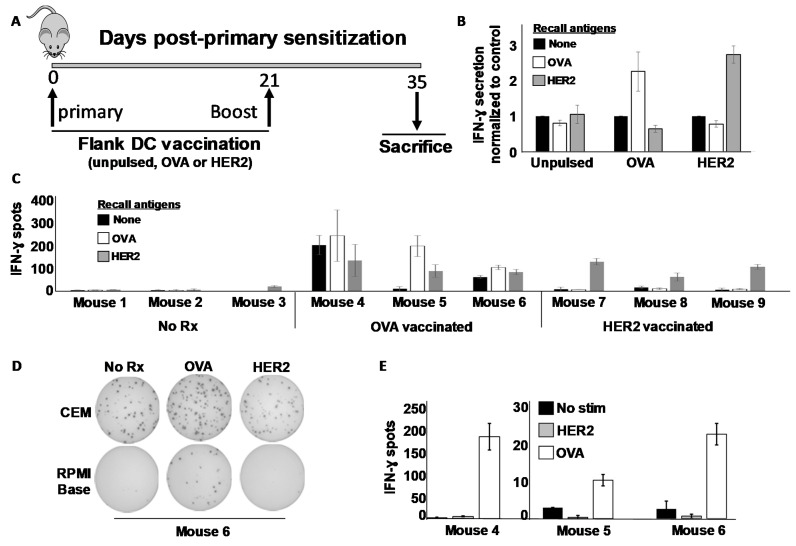
Murine DCs cultured in a serum-free medium can prime in vivo against both MHC class I and class II-restricted antigens. (**A**) Schema of sensitization protocol. Balb/c mice were vaccinated subcutaneously in the flank on day 0 with 1 × 10^6^ SFM-DC loaded with either OVA (ISQAVHAAHAEINEAGR) MHC class II peptide, rat ErbB2/HER2 (TYVPANASL) MHC class I peptide or left unpulsed. Mice received an identical booster on day 21, and on day 35 mice were sacrificed and immune splenocytes subjected to analysis. (**B**) IFN-γ ELISA analysis of 24 h culture supernatants from immune splenocyte (three mice per treatment group) stimulated with various recall antigens. (**C**) IFN-γ ELISPOT analysis from stimulated splenocytes from individual mice. (**D**) Digital images of ELISPOT wells from representative mouse (#6) performed in either commercial ELISPOT culture medium (CEM; upper images) or base RPMI medium (lower images). (**E**) Enumeration of IFN-γ spots from splenocytes of vaccinated mice stimulated in base RPMI medium without added serum. Error bars for all panels indicate SEM.

We next sought to determine whether the SFM-DCs could sensitize T cells to either MHC class I- or MHC class II-restricted antigens, choosing the rat ErbB2/HER-2 (rHER-2) protein sequence (i.e., TYVPANASL) previously identified as an H-2K^d^-restricted (i.e., MHC class I) CTL epitope often referred to as “p66” [24], and the well-characterized I-A^d^-restricted (i.e., MHC class II) Th epitope of ovalbumin (323–339) [26]. Mice were vaccinated in the flanks on day 0 and boosted once on day 21 (Figure 2A). Fourteen days after the last vaccination Day 35), mice were sacrificed and their splenocytes stimulated in vitro with various recall antigens, and IFN-γ response evaluated both by ELISA and ELISPOT assays. From ELISA analysis, it was clear that splenocyte cultures from OVA-vaccinated mice secreted IFN-γ when stimulated with OVA but not rHER-2 peptides (Figure 2B). Conversely, splenocytes from rHER-2-vaccinated mice secreted the cytokine in response to rHER-2 but not OVA peptides. Sham-vaccinated mice showed no such response to either peptide. Tandem ELISPOT analysis revealed a similar picture, but with one caveat; some OVA-vaccinated mice displayed an unusually high background not demonstrated by rHER-2 vaccinated mice (Figure 2C and Supplementary Materials Figure S3).

Because this background was only observed with splenocytes from OVA-sensitized mice, we considered the possibility that there was some OVA cross-reacting antigen in the commercial ELISPOT medium supplied with the kit. To test this hypothesis, we re-tested the same splenocytes (the excess of which had been cryopreserved at the time of the first assay), stimulating them with OVA and rHER-2 peptides in the same commercial ELISPOT medium used in Figure 2C and Supplementary Materials Figure S3, or alternatively in RPMI base medium with no serum additives, identical to that which was used for the ELISA. Interestingly, we replicated the high background in the commercial ELISPOT medium (sample Elispot wells for mouse #6 shown in Figure 2D), while obtaining good antigen specific recall responses with extremely low background using the base RPMI medium (Figure 2D,E). It thus appeared that the commercial Elispot medium contained a component that could stimulate OVA-immune splenocytes, but this issue could be circumvented using additive-free RPMI which, despite lack of added serum, could clearly support T cell viability and cytokine secretion during the brief 24 h ELISPOT culture period.

### 3.3. rErbB2/HER2 CTL Peptide-Pulsed DCs Cultured under Both Serum-Free and Serum-Replete Conditions Display Therapeutic Activity in a Mouse Model of HER2^pos^ Breast Cancer

After establishing that SFM-DCs could sensitize in vivo against known MHC-class I and MHC class II-restricted antigens, we decided to compare head-to-head the therapeutic activity of these cells with standard FCS-DC. For this test, we chose a mouse model of breast cancer using the TUBO breast carcinoma line [23], which is derived from transgenic Neu-T mice of Balb/c (H-2^d^) background.

Neu-T mice overexpress rat ErbB2/HER2 in breast tissues and spontaneously develop breast cancer in all mammary glands [27]. TUBO was derived from such mice and can be propagated in culture and implanted orthotopically in Balb/c mice resulting in tumors that can exceed 200 mm^2^ typically by about day 30–40 post-implantation, providing enough time to test various therapies in established tumors.

**Figure 3:**
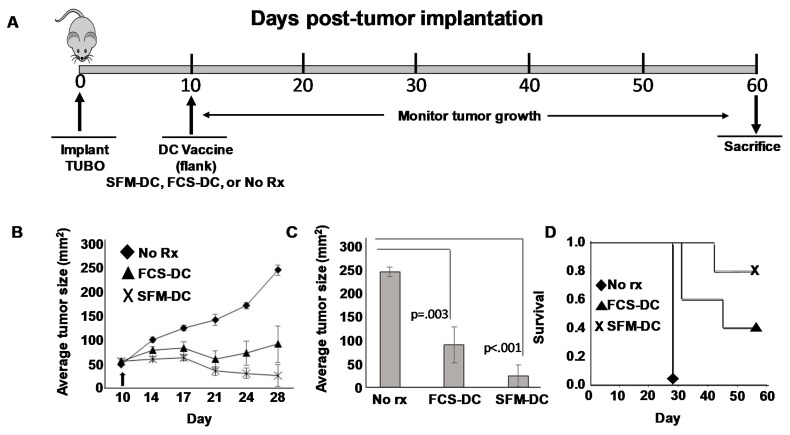
Comparison of efficacy of peptide-pulsed DCs cultured in serum-free versus serum-replete medium in a therapy model for HER2^pos^ breast cancer. (**A**) Schema of therapy protocol. On day 0, fifteen female Balb/c mice received 1 × 10^6^ orthotopically-implanted TUBO breast carcinoma cells. On day 10 mice were divided into three groups (five mice each) and vaccinated subcutaneously in the flank with rat ErbB2/HER2 class I peptide (TYVPANASL) pulsed either onto DCs cultured in X-Vivo 20 (SFM-DC) or in RPMI supplemented with 10% fetal calf serum (FCS-DC). Alternatively, mice were left untreated (No Rx). (**B**) growth curves of tumors over the course of 28 days. (**C**) Average tumor size comparison on day 28 with statistical analysis. (**D**) Kaplan–Meyer survival plot where vaccinated mice were followed for an additional 30 days. Failure to survive was defined as tumor size exceeding size of 200 mm^2^.

Mice bearing 10-day established TUBO orthotopically implanted tumors were divided into three groups. The first group was left untreated. The second group received a single subcutaneous (flank) injection of rHER-2 p66 (i.e., TYVPANASL) peptide-pulsed FCS-DCs. The third group received rHER-2 p66 peptide-pulsed SFM-DCs (Figure 3A). Tumor size was measured twice weekly and tumor area calculated. Growth kinetics curves (Figure 3B) showed that both FCS-DC and SMF-DC could suppress tumor growth compared with untreated mice. Statistical analysis was performed at the terminus of the experiment (when tumors in untreated control mice exceeded 200 mm^2^ size limits) at 28 days post-implantation (Figure 3C). Average tumor size for both treatment groups were significantly smaller than untreated mice. Nonetheless, despite a trend for smaller average tumors in the SFM-DC group compared with the FCS-DC group, the difference did not reach statistical significance.

We also evaluated therapy in this experiment by Kaplan–Meyer survival analysis, continuing to follow the vaccinated mouse groups and using attainment of tumor size exceeding 200 mm^2^ as an endpoint (Figure 3D). Once again, a clear trend was observed for enhanced survival for mice vaccinated with SFM-DC versus FCS-DC, but this difference did not prove to be statistically significant. We conclude from these studies that the therapeutic activity of SFM-DC was at least as good as FCS-DC, with some evidence that they may possibly be therapeutically advantageous.

### 3.4. Dendritic Cells Cultured in Serum-Free Medium Exhibit Modest Killer Function on TUBO Breast Carcinoma Cells

**Figure 4:**
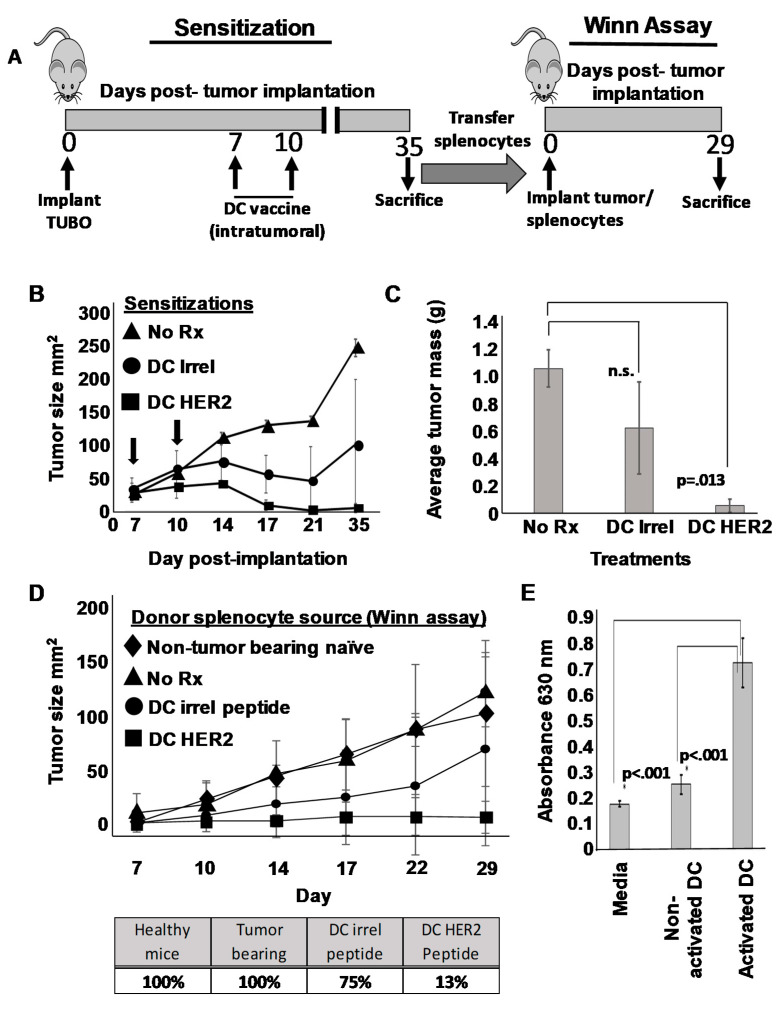
DCs possess a discrete anti-tumor activity that is not dependent on loading with tumor-associated antigen. (**A**) Schema of treatment protocol. On day 0, 15 female Balb/c mice were implanted orthotopically with 1 × 10^6^ TUBO breast carcinoma cells. On day 7, mice were divided into three groups of five each and received via intratumoral administration 1 × 10^6^ SFM-DCs pulsed with either rat Erb/B2/HER2 class I peptide, TYVPANSL (DC HER2) or irrelevant peptide (DC Irrel). The third group was left untreated (No Rx). On day 10 mice received a second, identical inoculation. Tumor size was measured twice weekly until day 35, when all mice were sacrificed, pooled splenocytes from each of the three groups were admixed with cultured TUBO cells, and in a Winn-type assay, injected orthotopically into fresh naïve mice. A fourth control group with TUBOs combined with naïve mouse spleens was also included. Tumor growth was measured twice weekly, and all mice were sacrificed 29 days after tumor implantation. (**B**) Tumor growth curves (sensitization phase) for the three treatment groups. Arrows indicate the two intratumoral DC vaccinations. (**C**) Average mass of tumors excised from sacrificed mice on day 35 for each treatment group. (**D**) Tumor growth curves (upper panel) from the Winn-type assay. Percentage of mice (lower panel) that developed tumors in Winn assay. (**E**) Culture supernatants from DCs either treated with ODN1826 and LPS (activated DC) or not (Non-activated DC) were removed 24 h later and added at a 1:1 ratio with cultured TUBO cells. After 72 h Alamar Blue dye was added and optical density of supernatants determined several hours later at 630 nm.

A number of investigators have reported that some DC preparations can exert a direct suppressive effect on tumor cells that some have referred to as “killer DC” function [28,29]. In an attempt to gain insight as to whether the SFM-DC possessed such activity, we designed experiments where DCs would be introduced directly into tumors. TUBO cells were implanted orthotopically and 7 days later (when tumors were palpable), therapy commenced consisting of vaccination with SFM-DCs pulsed with either rHER-2 p66 class I peptide, or an irrelevant antigen (i.e., non-tumor peptide). A booster vaccine was given 3 days later (i.e., day 10) (Figure 4A). A third group of tumor-bearing mice was left untreated. As expected, with untreated mice TUBO cells grew progressively, and by day 35 mice would have to be euthanized due to large, progressively growing tumors (Figure 4B). In contrast, intratumoral vaccination with rErbB2/HER2 peptide-pulsed DCs resulted in potent suppression of tumor growth. As expected, mice receiving intratumoral vaccine with SFM-DCs pulsed with irrelevant peptide also showed retarded average tumor size across time, consistent with the notion that DCs themselves could exert an incomplete, yet detectable anti-tumor effect that was not dependent on a pulsed, tumor-associated antigen. At the terminus of the experiment (day 35 post-implantation), all mice were sacrificed. Tumors were removed, and extraneous connective tissues carefully cut away from each mass. The resulting tumors were then blotted dry, weighed, and average tumor mass determined (Figure 4C). As expected, these results were closely congruent with the tumor caliper measurement studies with statistically significant (*p* < 0.013) suppression of average tumor mass in rHER-2 vaccinated mice and a more modest suppression of tumor mass in mice vaccinated with irrelevant peptide-pulsed DC.

If SFM-DCs pulsed with irrelevant peptide exerted only a direct “killer” effect on tumor cells, then no transferrable immunity should be contained in splenocytes from mice vaccinated with these cells. To test this, single cell suspensions from spleens obtained from mice from all three treatment groups, as well as unvaccinated, non-tumor bearing controls were individually mixed with freshly harvested cultured TUBO cells, and, in a Winn-type assay, injected subcutaneously into fresh naïve mice (Figure 4A). Tumor outgrowth was then monitored and recorded. As expected, TUBO cells mixed with splenocytes from either healthy or unvaccinated tumor-bearing mice all formed tumors that grew with characteristic rapidity when injected into naïve mice (Figure 4D upper panel). In contrast, mice implanted with TUBO cells mixed with rHER-2 immune splenocytes displayed profoundly suppressed average tumor size throughout the monitoring period. Contrary to our expectations, splenocytes from mice vaccinated intratumorally with SFM-DCs pulsed with irrelevant peptide displayed a modest, yet notable reduction in average tumor size. We also evaluated this experiment by observing the percentage of mice developing tumors for each treatment group (Figure 4D lower panel). All mice receiving TUBOs mixed with normal or untreated tumor-bearing mouse splenocytes formed progressively growing tumors. In contrast only one of eight mice (i.e., 13%) implanted with TUBO mixed with rHER-2 immune splenocytes formed tumors. However, mice inoculated with TUBO mixed with splenocytes from irrelevant peptide-pulsed SFM-DC formed tumors in 70% of mice. By either analysis, these results suggested that modest transferrable immunity was indeed induced even when DCs were not pulsed with tumor peptide antigens.

These unexpected results called into question whether the SFM-DCs actually possessed any direct anti-tumor activity. We therefore attempted to shed light on this question with an in vitro experiment. To test whether any soluble factors produced by DC could have anti-tumor activity, we collected culture supernatants from either activated or non-activated SFM-DCs and tested the supernatants for their capacity to suppress TUBO cells in an Alamar Blue assay (Figure 4E). As can be seen, neither normal media nor supernatants from unactivated stage II culture of SFM-DC had an appreciable effect on the ability of TUBO cells to metabolize the Alamar Blue dye. In contrast, supernatants from activated SFM-DCs significantly reduced metabolic activity in TUBO cultures. We next attempted to determine which soluble factor, or combination of factors, might be responsible for this observed anti-tumor cell activity. We therefore utilized an antibody protein array capable of detecting a panel of 97 common cytokines, including those associated with inflammation (Supplementary Materials Figure S4). Of these 97 tested, eight were strongly up-regulated by DC activation using a combination of LPS and ODN1826. These included IL-12 p70, p40, RANTES, CXCL1, Eotaxin-2, IL-6, MIP-1α, and MIP-2. We then added combinations of recombinant versions of these cytokines to TUBO cells to determine if any of these detected products were responsible for the observed effects. We found that no combination of these cytokines tested could account for the observed effects of the culture supernatants (not shown). These results suggest that activated SFM-DC produce soluble factor(s) that can negatively impact TUBO cells in vitro, but that activity seen in vivo cannot be distinguished from the ability of intratumorally-placed DCs to induce weak transferrable immunity (implying lymphocyte sensitization) even when they are not pulsed with tumor-associated antigen.

### 3.5. Identification of Novel Helper T Cell Epitopes That Display Strong Therapeutic Activity in Mouse Breast Cancer Model When Used as a Component of a Th1-Polarizing Dendritic Cell Vaccine

**Figure 5:**
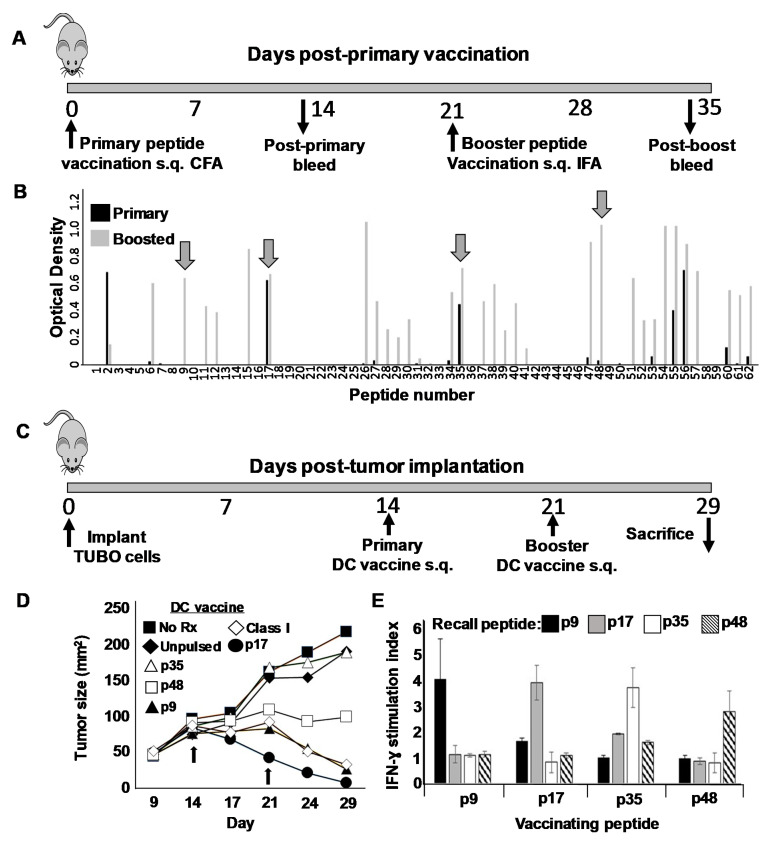
Identification of Helper T cell epitopes on rat ErbB2/HER2. (**A**) Schema of peptide vaccination protocol. On day 0 female Balb/c mice were vaccinated subcutaneously (s.q.) with pools of 5–6 peptides (30 µg each) based on the rat ErbB2/HER2 extracellular domain sequence and emulsified in Freund’s Complete Adjuvant. On day 21 mice received an identical booster immunization in Freund’s Incomplete adjuvant. Mice were bled 12–14 days after each vaccination (about day 14 and 35) to obtain immune sera. (**B**) ELISA analysis for IgG production against individual pool peptides. Four peptides (indicated by arrows) were selected for further study: p9; QEVQGYMLIAHNQVKRVPLQR, P17; GNPQLCYQDMVLWKDVFRKN, p35; PCARVCYGLGMEHLRGARAI and p48; LIHRNAHLCFVHTVPWDQLF). (**C**) Schema for therapeutic DC vaccination with novel putative Th peptides. Female Balb/c mice (five per treatment group) received 1 × 10^6^ TUBO breast carcinoma cells implanted orthotopically on day 0. On days 14 and 21 mice received subcutaneous flank vaccination with 1 × 10^6^ SFM DCs pulsed with putative Th peptides p9, p17, p35 and p48. Other groups received DCs pulsed with rat ErbB2/HER2 CTL peptide TYVPANASL (Class I), or unpulsed DC. A final group of tumor-bearing mice was left untreated (No Rx). Mice were monitored for tumor size twice weekly and were sacrificed on day 29. (**D**) Tumor growth curves for mice receiving various indicated SFM-DC vaccines. (**E**) Splenocytes from sacrificed mice vaccinated with SFM-DC pulsed with either p9, p17, p35 or p48 were cultured in the presence of various recall peptides and 24 h supernatants analyzed for IFN-γ via ELISA analysis. Error bars indicate SEM.

Whereas historically, CD8^pos^ cytotoxic T cells have been regarded as the primary T cell arm for anti-tumor immunity, more recently the contributions of Th cells are being appreciated [30]. We therefore next sought to identify novel Th epitopes on rErbB2/HER2 and test these peptides in therapy models compared with the well-characterized rErbB2/HER2 CTL epitope p66. We began these studies by constructing a peptide library consisting of a series of 62 peptide 20-mers each overlapping by 10 amino acids and spanning the entire extracellular domain of rErbB2/HER2 (Supplementary Materials Figure S5). Because we expected only a minority of these 62 peptides to contain Th epitopes, we devised a screening step to economically filter out uninteresting peptides. To accomplish this, we partitioned the 62-peptide library into twelve pools of five or six peptides each, emulsified the individual pools in complete Freund’s adjuvant, and vaccinated mice subcutaneously in the flanks (Figure 5A). After 21 days, mice received a booster immunization with the same peptide pool emulsified in incomplete Freund’s. Blood was removed from the mice 12–14 days after each vaccine and the presence of serum antibodies (IgG) against individual peptides within each of the vaccinating pools determined via ELISA analysis (Figure 5B). We reasoned that since IgG secretion by B cells requires T cell help, an individual peptide’s capacity to induce IgG production likely indicated the presence of an embedded Th epitope stimulatory to CD4^pos^ T cells. We observed that peptides fell into three distinct categories: first, those that provoked little or no IgG on either primary sensitization or boosting (e.g., 3–5, 19–25, 42–46, etc.); second, those that demonstrated weak or no IgG on primary sensitization, but became strong after one boost (e.g., peptides 6, 9, 26, 48 and 57); and finally, those that induced a strong response after only one sensitization (e.g., peptides 17, 35 and 56). Naturally, the non-immunogenic peptides in the first category were immediately dismissed as unworthy of further study. We then selected four putative epitopes for further analysis: two each from the latter categories, including p9 and p48 from category two, and p17 and p35 from category three.

We next tested the therapeutic activity of each of the four putative Th epitopes in mice bearing 14-day orthotopically placed TUBO breast carcinoma tumors. Tumor-bearing mice were divided into seven groups of five mice each, and received either no treatment, unpulsed SFM-DCs, SFM-DCs pulsed with either p9, p17, p35 or p48, or finally SFM-DCs pulsed with the rErbB2/HER2 class I-restricted CTL epitope p66 (TYVPANASL). One week later, mice received an identical booster vaccine (Figure 5C). Animals were monitored daily and tumor measurements taken twice weekly (Figure 5D). Tumors in mice that received either no treatment or unpulsed SFM-DCs grew progressively at approximately the same rate. Interestingly, we observed great diversity in the therapeutic activity of the putative Th epitope peptides. Mice vaccinated with p35, for example, displayed no measurable retardation in growth kinetics compared with untreated mice or those receiving unpulsed SFM-DC. In contrast, p17 showed powerful therapeutic activity (*p* < 0.001 compared with unpulsed DC) which was noted even before the booster vaccination and induced rapid regression of the tumor in all mice, leading to apparent cures. Of somewhat less activity was p9, which also induced regressions, although these were not apparent until after boosting (*p* < 0.001 compared with unpulsed DC). The requirement for boosting to induce a therapeutic response with p9 was consistent with the observed requirement for boosting both to observe a strong antibody response against this peptide (Figure 5A) as well as the ability to consistently detect IFN-γ recall responses from immune splenocytes (not shown). Peptide p48 with the tested vaccine schedule seemed to arrest tumor growth without actual regression, but the trend toward suppression of growth was not statistically significant compared with unpulsed DC. The peptide containing the CTL epitope had moderate activity roughly comparable to p9. At the termination of this therapy experiment, mice from each group were sacrificed and single cell suspensions made from spleens, which were then stimulated for 24 h with each of the four putative Th epitopes used for vaccination. Supernatants from stimulated cells were then evaluated for the presence of IFN-γ. Despite the dramatic differences in therapeutic activity between the peptides, we observed similar IFN-γ recall responses (on the order of at least 2 ng/mL) for each vaccinating peptide (Figure 5E).

### 3.6. Mice Vaccinated with DCs Loaded with Putative Th Epitope-Containing Peptides Display Recall Responses Characterized by Th1 Cytokines from CD4^pos^ but Not CD8^pos^ T Cell Populations

**Figure 6:**
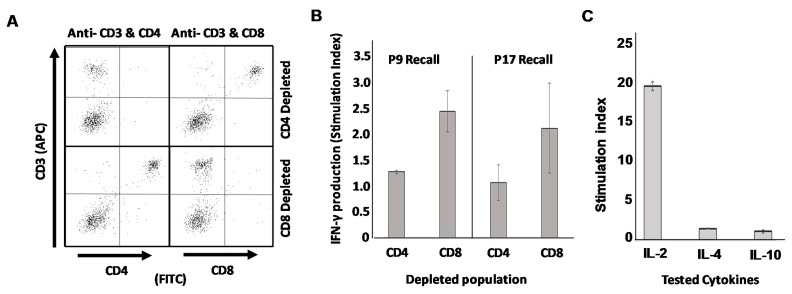
Recall responses to identified peptide epitopes are mediated by CD4^pos^ T cells and not CD8^pos^ T cells. Mice immunized with activated DCs pulsed with either p9 or p17 were sacrificed and single cell suspensions made of their spleens 16 days later. Cells were then subjected to magnetic bead separation to remove either CD4^pos^ or CD8^pos^ cells from total splenocyte populations. (**A**) Representative quality control FACS analysis of separated cells from p17 vaccinated mice demonstrating successful specific removal of either CD4^pos^ or CD8^pos^ populations. (**B**) CD4^pos^- and CD8^pos^- depleted splenocyte populations were each stimulated in vitro with vaccinating peptides and 24 h supernatants analyzed for IFN-γ production by ELISA. (**C**) Peptide-stimulated 24 h culture supernatants from unfractionated splenocytes of p17-vaccinated mice were analyzed for IL-2, IL-4 and IL-10 via ELISA.

Finally, we wished to definitively prove that the epitopes we discovered were in fact true Th epitopes recognized exclusively by CD4^pos^ T cells rather than CD8^pos^ CTL. To accomplish this, we once again vaccinated mice with SFM-DCs pulsed with either putative Th epitope P9 or p17, the only two peptides that induced statistically significant suppression of TUBO growth compared with unpulsed DC controls (Figure 5D). At 17 days post-vaccination mice were sacrificed, and single cell suspensions made of their spleens. These cells were then subjected to immuno-depletion using paramagnetic beads to remove either CD4^pos^ T cells or CD8^pos^ T cells from whole splenic populations. Splenocytes depleted of either population were then stimulated with the vaccinating peptide to determine which population of T cells was responsible for recall production of IFN-γ. Sample quality control (Figure 6A) consisting of flow cytometry analysis (CD3 versus CD4 and CD3 versus CD8) demonstrated highly efficient removal of either CD4^pos^ or CD8^pos^ populations from murine splenocytes for both p9- and p17-vaccinated mice. When supernatants from population-depleted, peptide-stimulated splenocytes were evaluated for the presence of IFN-γ, it was apparent that depletion of CD4^pos^ but not CD8^pos^ T cells resulted in the loss of IFN-γ recall responses to peptide (Figure 6B). We therefore conclude that these novel epitopes with therapeutic activity represented by p9 and P17 are true Th epitopes recognized by CD4^pos^ T cells.

In a final experiment, supernatants from peptide-stimulated splenocytes of p17 SFM-DC-vaccinated mice were analyzed via ELISA for additional cytokines including IL-2 (also associated with Th1 cells), IL-4 (associated with Th2 cells) and IL-10 (associated both with Th2 cells and T_reg_). Strong IL-2 recall response to peptide was observed, but no appreciable IL-4 or IL-10 was detected (Figure 6C). Additionally, protein antibody chip analysis of the same supernatants for various IL-17 isoforms did not detect any of this Th17-associated cytokine (data not shown). It thus appears that these SFM-DCs activated with the combination of ODN1826 and LPS induce highly polarized Th1 responses in vivo, with no detectable contribution of other common Th phenotypes.

## 4. Discussion

Dendritic cells remain a viable platform for developing immune-based therapies against cancer [31,32]. The standards for preparing DCs for use in humans include culture in serum-free media for reasons of safety and reproducibility. For studies using animal models, it is also reasonable to develop similar serum-free methods, not only to harmonize culture conditions so that translation to human clinical trials is facilitated, but also because the plethora of xenoantigens found in fetal calf serum, if processed and presented by the DC, can potentially confound interpretation of immunological studies. Other investigators have recognized this problem and developed serum-free culture conditions for murine DCs [15,33], but our studies are the first to adapt the two-stage protocol developed previously by Cohen et al. [12] to serum-free conditions. In addition, our work more completely characterizes the functional qualities of these DCs including their ability to serve as therapeutic agents in a mouse model of HER-2^pos^ breast cancer.

When comparing different culture media, we found that each of several commercial serum-free preparations performed about equally well in allowing expansion of DC precursors in “stage I” (i.e., Flt-3/IL-6-supplemented) culture and were not statistically different from “standard” FCS-supplemented RPMI. In fact, the only medium that completely failed to support growth and expansion of BM DC precursors in stage I was RPMI base medium with no added serum. It should be noted that even commercial SFM is not entirely protein-free and does contain additives such as albumin as well as other proteins. They therefore represent a reduction of xenoantigens to which the DCs are exposed during culture, but not a complete elimination. Although not the case for 6-day stage I culture, we did find that the relatively brief overnight stage II culture in GM-CSF and IL-4, including peptide-loading, and activation with ODN1826 and LPS could be accomplished in base RPMI medium (i.e., no added serum). We chose to use the base RPMI during stage II to absolutely minimize DC opportunity to acquire any antigen other than the intended peptides for in vivo sensitization studies and therapy models.

The cells produced in this manner displayed outstanding DC performance characteristics including high IL-12p70 secretion upon activation, and robust expression of markers associated with mature DCs. They also showed functional capacity that included the ability to sensitize in vivo against both MHC class I (rHER-2 p66) and MHC class II (OVA) antigens, as well as therapeutic activity in a murine model of HER-2^pos^ breast cancer, when pulsed with novel Th epitopes from the rErbB2/HER2 ECD. The DCs appeared to strongly polarize T cells toward the Th1 phenotype, as evidenced by the secretion of IFN-γ and IL-2 with the corresponding absence of cytokines associated with other Th phenotypes. Interestingly, we found that cultured splenocytes from some mice sensitized against OVA displayed unusually high IFN-γ secretion even when unstimulated with specific recall antigen. This was seen only with OVA but not rHER-2 sensitized mice, and was observed only in the ELISPOT assay, not the ELISA assay. Because the ELISPOT assay utilized a proprietary culture medium included with the kit, we hypothesized that a component in the ELISPOT medium must, through some cross-reactivity, elicit a spurious recall response from the OVA-sensitized splenocytes. Consistent with this explanation, we eliminated the high background cytokine secretion in unstimulated cells while preserving recall response in the presence of added OVA when culture medium for splenocyte assays was switched to base RPMI without added serum. We searched the published literature for any report of cross-reactivity between ovalbumin and serum components, and interestingly, a decades-old study described a serological cross-reaction between ovalbumin preparations and bovine serum albumin [34]. However, upon thorough investigation the cross-reaction seemed to be against an impurity of the crystalized OVA. Since the OVA used in our studies is a synthetic peptide, this cannot be the explanation for our findings. Nonetheless, our observation represents a cautionary tale: commercial medium that typically comes with such kits can on occasion contain components that will induce spurious or cross-reacting signals in assayed cells. It also suggests the use of base medium without added serum proteins as a workable solution that provides much lower background in short term cultures (<24 h) without much loss of signal.

In terms of functional activity assessed in a murine therapy model of HER-2^pos^ breast cancer, a side-by-side comparison the SFM-DC showed activity that was at least on par with FCS-DC. Although growth curves comparing average tumor size between treatment groups appeared to show a great distinction between mice vaccinated with either of the two DC preparations, with the SFM-DC displaying an apparent advantage, the difference did not rise to statistical significance. One likely reason for this was the great variability in responses from individual treated mice within treatment groups. Therapeutic responses often presented as an “all or nothing” proposition, with individual tumors regressing to an apparent cure or continuing to grow until reaching a size that required euthanasia. Nonetheless, only two out of five mice immunized with FCS-DC achieved complete regression of tumors while four of five mice receiving SFM-DC showed such dramatic cures. We therefore conclude that SFM-DC are at least as good functionally as those derived from traditional serum-replete conditions, with strong trends supporting the SFM-DC as the better choice for maximal therapeutic activity when used as vaccines.

We also briefly examined a lesser-explored functional capacity for the SFM-DC attributed to some DC preparations, that is, putative “killer DC” function [28,29]. In vitro studies seemed to indicate that activated DC secreted a soluble factor or factors that suppressed metabolic activity in TUBO breast carcinoma cells as assessed by the Alamar Blue assay. Protein antibody array chip analysis identified a restricted set of pro-inflammatory factors produced in quantity by the activated DCs, but we could not reproduce the metabolic suppression of TUBO cells with recombinant versions from this set of cytokines in any combination (not shown). Other investigators have demonstrated that different populations of DC can secrete products with tumoricidal activity including TRAIL, perforin and granzyme B and NO [35,36]. Among these, the cytokine antibody array assay we performed was capable of detecting only Granzyme B, for which we observed no enhancement upon DC activation. Therefore, we now consider these other three factors the likeliest source for our observed anti-tumor activity. Interestingly, activated DCs pulsed with irrelevant peptide and injected directly into established TUBO tumors suppressed tumor growth to a level between that of untreated mice and those immunized intratumorally with HER-2 CTL peptide-pulsed SFM-DC, initially suggesting that some in vivo killer DC activity was detected. However, when splenocytes from these mice were tested in a Winn-type assay, they unexpectedly showed some weak anti-tumor activity, calling into question this interpretation by suggesting that transferable anti-tumor lymphocytes were being induced by DCs containing no exogenously-pulsed tumor antigens. There are at least two explanations for these observations. The first is that the SFM-DCs show no real killer activity in vivo, and the suppressed tumor growth seen when irrelevant peptide-pulsed DCs are injected intratumorally arises from the fact that the DCs pick up native tumor antigens in situ and sensitize lymphocytes against them. The second is that the observed effects are a combination of some direct killer DC function coupled with lymphocyte sensitization of endogenously-acquired tumor antigen. Intratumorally-administered SFM-DCs may do some killing and also pick up endogenous antigens in situ for presentation to lymphocytes, which in turn contribute to anti-tumor activity. The possible killer DC effect is worthy of further exploration but is beyond the scope of the current study. It should be noted, however, that the intratumoral administration of tumor peptide-pulsed DCs is currently being systematically studied as a possibly superior method of vaccine delivery (K. Kodumudi, manuscript in preparation).

Whereas CD8^pos^ CTL have been traditionally considered to be primary effectors in anti-tumor immunity, the last two decades have ushered in a steady stream of studies indicating the potential of CD4^pos^ T cells for controlling malignancies. This has been particularly the case for breast cancer, and our own group has performed several DC-based vaccine clinical trials for HER-2^pos^ breast cancer [20,37,38,39] using promiscuous HLA class II binding HER-2 peptides [38,40] as immunogens. Modeling immunotherapies for HER2-positive disease would therefore be facilitated by identifying optimal MHC class II-restricted CD4 T cell epitopes for rHER-2. Using a rat HER-2 ECD overlapping peptide library, we settled on four putative CD4 T cell epitopes contained in peptide 20-mers that were capable of eliciting strong IgG responses when administered in Freund’s adjuvant, and strong IFN-γ recall responses from immune splenocytes when peptide-pulsed DCs were used as vaccines.

Interestingly, whereas all four putative CD4 T cell epitopes sensitized for comparable IFN-γ recall responses when pulsed onto SFM-DCs, there were great differences in therapeutic activity observed when SFM-DCs pulsed with each individual peptide were compared for activity in a therapy model for established rHER-2^pos^ breast cancer using orthotopically implanted TUBO breast carcinoma cells (Figure 5B). For example, p17 elicited very strong therapeutic effects leading to complete cures in 5/5 immunized mice. In contrast, p35-pulsed DCs had no discernable effect, with its growth curves virtually overlapping with both untreated mice and mice sham immunized with unpulsed DCs. A likely explanation is that the epitope contained in p35, due to differential antigen processing of the native rHER-2, is not available for presentation by MHC class II-expressing cells resident within the tumor bed. Therefore, there is nothing for p35-sensitized CD4^pos^ T cells to “see” at the site of disease and thus their effector functions are not triggered locally. The epitope represented by p17, in contrast, is probably appropriately processed and presented, and p17-sensitized T cells are thereby able to be activated at or near tumor beds. The observation that different T cell epitopes fall into hierarchies based on antigen processing and other factors has been extensively described previously [39,41].

The notion that all four identified peptide 20-mers are legitimate CD4^pos^ T cell epitopes rather than CD8^pos^ CTL epitopes is consistent with the fact that 20mers are too large to fit in the peptide binding groove of MHC class I molecules, which usually can only accommodate peptides of much shorter lengths [40,42] and with our evidence that these 20-mers provoke strong IgG antibody responses, which generally require CD4^pos^ T cell help. Nonetheless, it is formally possible that the peptides could be broken down into smaller fragments capable of interacting with MHC class I molecules. Since we wished to determine with greatest confidence that these identified epitopes were recognized by CD4^pos^ but not CD8^pos^ T cells, we performed magnetic bead depletions of either CD4^pos^ or CD8^pos^ T cells from whole splenic populations of mice sensitized with DCs pulsed with our two best peptides (in terms of therapeutic performance), p9 and p17. We achieved very efficient depletions of each population and demonstrated that IFN-γ recall activity was found concentrated in the splenocyte preparations depleted of CD8^pos^ cells but not in those depleted of CD4^pos^ cells. We conclude therefore that the identified peptide 20-mers contain legitimate CD4 T cell epitopes.

## 5. Conclusions

A serum-free modification of the method reported by Cohen et al. [12] produced dendritic cells with yields, phenotype and function comparable to those cultured in medium supplemented with fetal calf serum. These functions included the ability to sensitize in vivo against both MHC class I and MHC class II-restricted peptides and the ability to generate therapeutically-meaningful immune responses in a mouse model of HER2^pos^ breast cancer using a known rat ErbB2/HER2 CTL epitope peptide. Furthermore, epitope mapping studies of rat ErbB2/HER2 led to the discovery of several novel helper T epitopes, a subset of which also elicited strong Th1-dominated responses and therapeutic activity in the murine breast cancer model. Identification of Th epitopes on other common oncodrivers and their inclusion in Th1-polarizing DC vaccines may be a fruitful avenue for developing DC-based cancer vaccine models for eventual translation into human trials.

## Data Availability

All of the data derived in this study are presented in the manuscript and in Supplementary Figures S1–S3.

## Supplementary Materials

The following are available online at https://www.mdpi.com/article/10.3390/vaccines9091037/s1, Figure S1. Digital images of Stage II cultured cells taken 4 hours after activation with LPS and ODN1826 taken with 20x objective using Zeiss Primovert microscope equipped with an Axiocam model 105 color-capable digital camera. Figure S2. Flow cytometry analysis of Stage II DC that completed stage I culture in various serum-free media (expressed as histograms). Stage II cultured DC were activated with a combination of ODN1826 and LPS, harvested and stained 24h later with fluorochrome-conjugated antibodies against I-Ad, CD11b, CD40, CD80 and CD86 (black traces). Gray traces represent isotype-matched controls. “FBS”, RPMI medium supplemented with 10% fetal calf serum; “HL-1”, RPMI medium supplemented with HL-1 serum substitute; “M-SFM”, Macrophage Serum-Free Medium; X-V20, X-Vivo 20 serum-free medium. Figure S3. Representative IFN-γ ELISPOT images of stimulated immune splenocytes from individual mice. Splenocytes from mice vaccinated with SFM-DC pulsed with either egg ovalbumin class II peptide (OVA) or rat ErbB2/HER2 class I peptide (HER2) or from untreated mice (none) were stimulated with recall antigen (OVA, HER2 or no stimulation [None]) in a IFN-γ ELISPOT assay. Figure S4. Analysis of products secreted by cultured DCs. Supernatants from cultured activated Stage II murine SFM-DCs either treated with ODN1826 and LPS (activated) or left untreated (unactivated) were collected and analyzed via protein antibody array for the expression of 97 cytokines and chemokines associated with inflammation. Boxes indicate products produced at higher levels by activated DCs. Figure S5. Sequences of all peptides in rat HER2 overlapping peptide library. A peptide library was constructed based on the extracellular domain sequence of rat ErbB2/HER2. It consisted of 62 peptide 20-mers overlapping by 10 amino acids each Underlined peptides (P9, p17, p35 and p48) were the four focused on in main studies.
