# Supplementary material for: Murine Dendritic Cells Grown in Serum-Free Culture Show Potent Therapeutic Activity when Loaded with Novel Th Epitopes in an Orthotopic Model of HER2pos Breast Cancer
Source: Vaccines (Basel). 2021 Sep 18;9(9):1037. doi: 10.3390/vaccines9091037 (PMC8473293; doi:10.3390/vaccines9091037)
Supplement: Supplementary file 1 [file vaccines-09-01037-s001.zip › vaccines-1357723-supplementary.pdf]

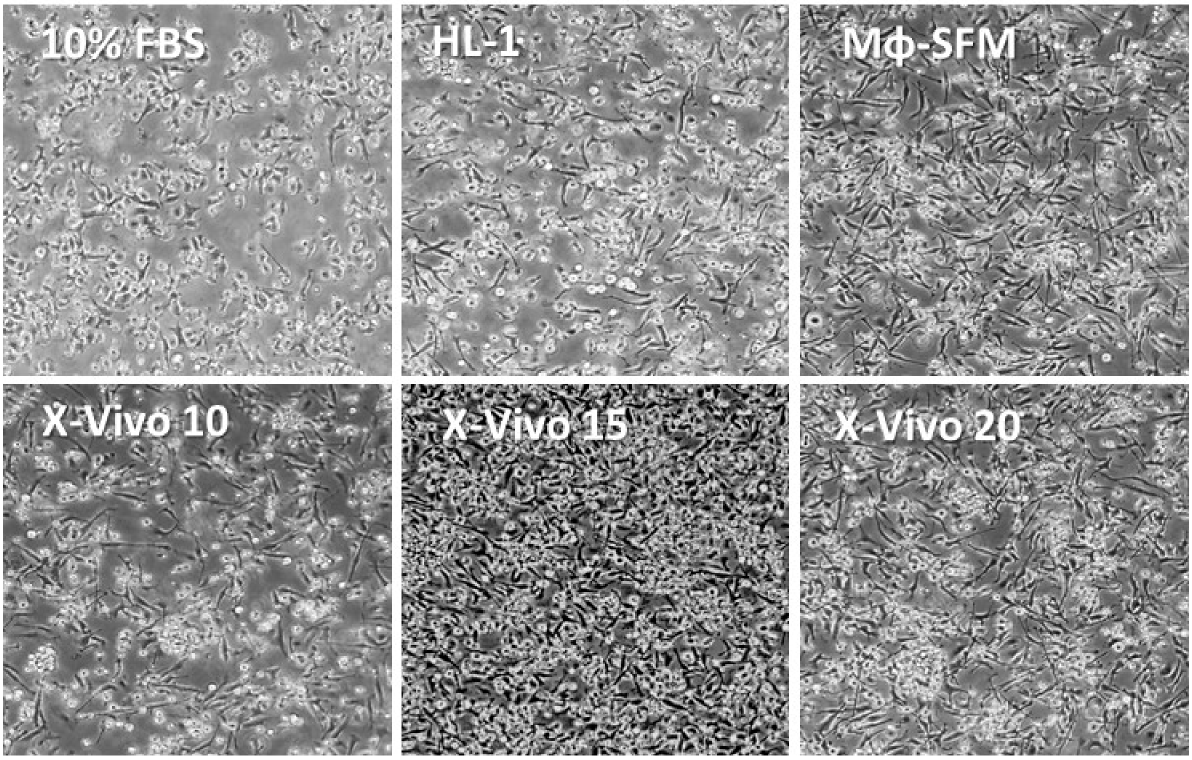

Supplementary Figure S1. Digital images of Stage II cultured cells taken 4 hours after activation with LPS and ODN1826 taken with 20x objective using Zeiss Primovert microscope equipped with an Axiocam model 105 color-capable digital camera.

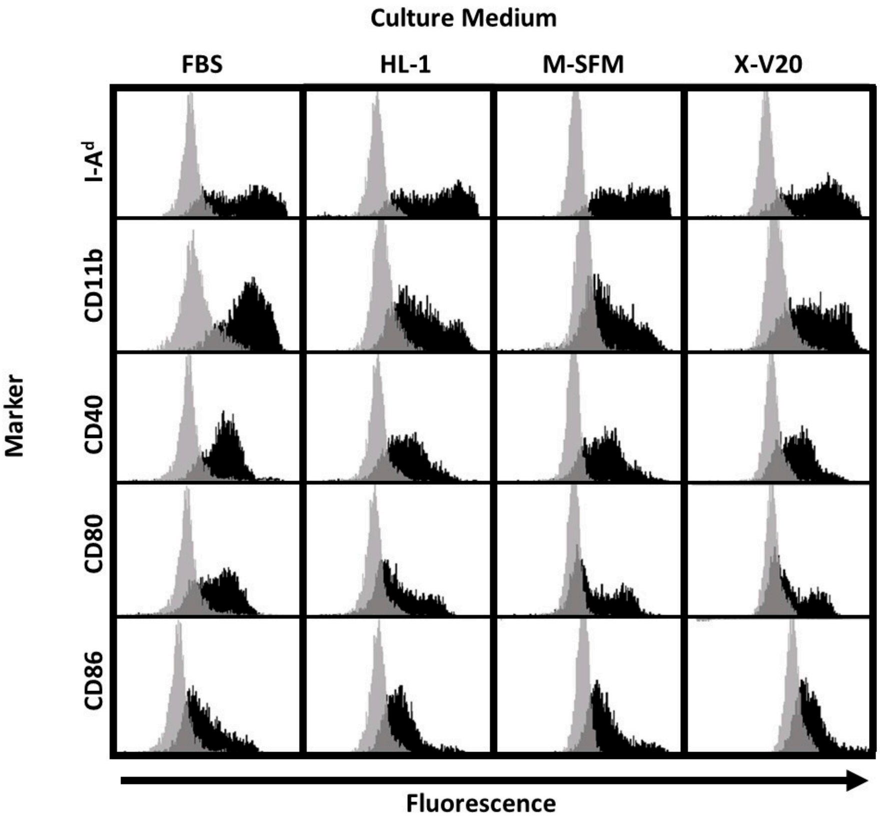

Supplementary Figure S2. Flow cytometry analysis of Stage II DC that completed stage I culture in various serum-free media (expressed as histograms). Stage II cultured DC were activated with a combination of ODN1826 and LPS, harvested and stained 24h later with fluorochrome-conjugated antibodies against I-A^d, CD11b, CD40, CD80 and CD86 (black traces). Gray traces represent isotype-matched controls. "FBS", RPMI medium supplemented with 10% fetal calf serum; "HL-1", RPMI medium supplemented with HL-1 serum substitute; "M-SFM", Macrophage Serum-Free Medium; X-V20, X-Vivo 20 serum-free medium.

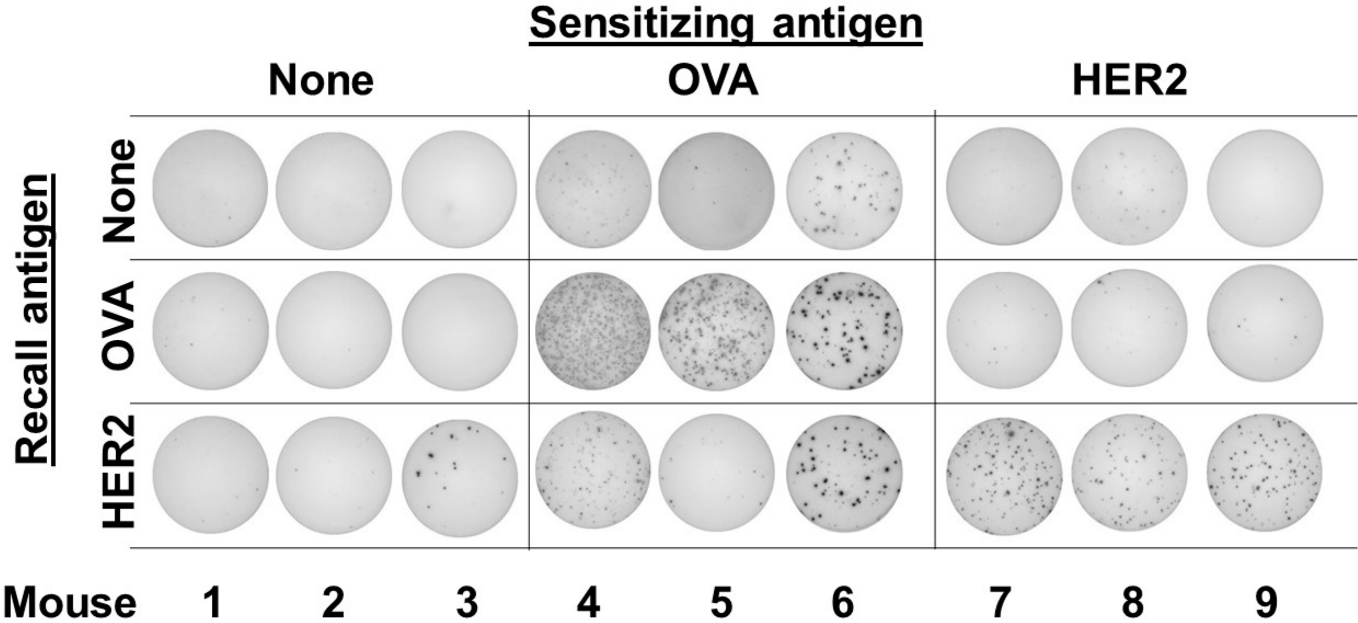

Supplementary Figure S3. Representative IFN-γ ELISPOT images of stimulated immune splenocytes from individual mice. Splenocytes from mice vaccinated with SFM-DC pulsed with either egg ovalbumin class II peptide (OVA) or rat ErbB2/HER2 class I peptide (HER2) or from untreated mice (none) were stimulated with recall antigen (OVA, HER2 or no stimulation [None]) in a IFN-γ ELISPOT assay.

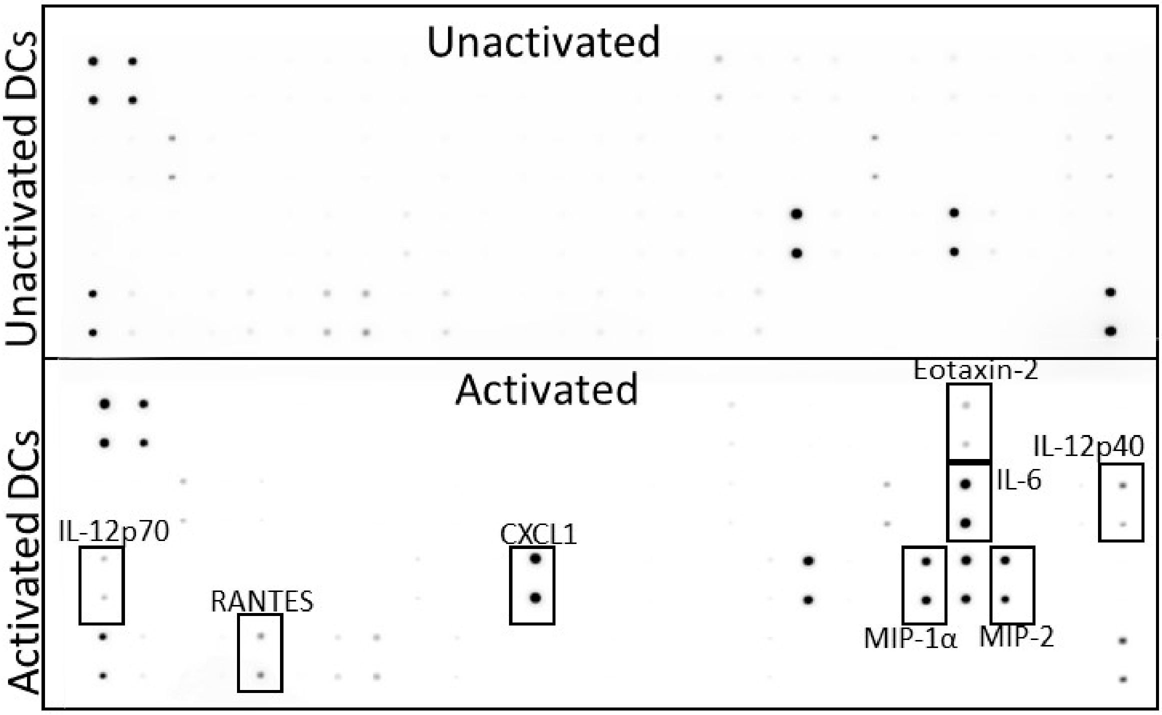

Supplemental Figure S4. Analysis of products secreted by cultured DCs. Supernatants from cultured activated Stage II murine SFM-DCs either treated with ODN1826 and LPS (activated) or left untreated (unactivated) were collected and analyzed via protein antibody array for the expression of 97 cytokines and chemokines associated with inflammation. Boxes indicate products produced at higher levels by activated DCs.

P1: ^{1}MIIMELAAWCRWGFLLALLP^{20}
P2: ^{11}RWGFLLALLPPGIAGTQVCT^{30}
P3: ^{21} PGIAGTQVCTGTDMKLRLPA^{40}
P4: ^{31} GTDMKLRLPASPETHLDMLR^{50}
P5: ^{41}SPETHLDMLRHLYQGCQVVQ^{60}
P6: ^{51} HLYQGCQVVQGNLELTYVPA^{70}
P7: ^{61}GNLELTYVPANASLSFLQDI^{80}
P8: ^{71}NASLSFLQDIQEVQGYMLIA^{90}
P9: ^{81}QEVQGYMLIAHNQVKRVPLQ^{100}
P10: ^{91}HNQVKRVPLQRLRIVRGTQL^{110}
P11: ^{101}LRIVRGTQLFEDKYALAVL^{120}
P12: ^{111}FEDKYALAVLDNRDPQDNVA^{130}
P13: ^{121}DNRDPQDNVAASTPGRTPEG^{140}
P14: ^{131}ASTPGRTPEGLRELQLRSLT^{150}
P15: ^{141}LRELQLRSLTEILKGGVLIR^{160}
P16: ^{151}EILKGGVLIRGNPQLCYQDT^{170}
P17: ^{161}GNPQLCYQDMVLWKDVFRKN^{180}
P18: ^{171}VLWKDVFRKNNQLAPVDIDT^{190}
P19: ^{181}NQLAPVDIDTNRSRACPPCA^{200}
P20: ^{191}NRSRACPPCAPMCKDNHCWG^{210}
P21: ^{201}PMCKDNHCWGESPEDCQILT^{220}
P22: ^{211}ESPEDCQILTGTICTSGCAR^{230}
P23: ^{221}GTICTSGCARCKGRLPTDCC^{240}
P24: ^{231}CKGRLPTDCCHEQCAAGCTG^{250}
P25: ^{241}HEQCAAGCTGPKHSDCLACL^{260}
P26: ^{251}PKHSDCLACLHFNHSGICEL^{270}
P27: ^{261}HFNHSGICELHCPALVTYNT^{280}
P28: ^{271}HCPALVTYNTDTFESMHNPE^{290}
P29: ^{281}DTFESMHNPEGRYTFGASCV^{300}
P30: ^{291}GRYTFGASCVTACPYNYLST^{310}
P31: ^{301}TACPYNYLSTEVGSCTLVCP^{320}
P32: ^{311}EVGSCTLVCPPNNQEVTAED^{330}
P33: ^{321}PNNQEVTAEDGTQRCEKCSK^{340}
P34: ^{331}GTQRCEKCSKPCARVCYGLG^{350}
P35: ^{341}PCARVCYGLGMEHLRGARAI^{360}
P36: ^{351}MEHLRGARAITSDNVQEFDG^{370}
P37: ^{361}TSDNVQEFDGCKKIFGSLAF^{380}
P38: ^{371}CKKIFGSLAFLPESFDGDPS^{390}
P39: ^{381}LPESFDGDPSSGIAPLRPEQ^{400}
P40: ^{391}SGIAPLRPEQLQVFETLEEI^{410}
P41: ^{401}LQVFETLEEITGYLYISAWP^{420}
P42: ^{411}TGYLYISAWPDSLRDLSVFQ^{430}
P43: ^{421}DSLRDLSVFQNLRIIRGRIL^{440}
P44: ^{431}NLRIIRGRILHDGAYSLTLQ^{450}
P45: ^{441}HDGAYSLTLQGLGIHSLGLR^{460}
P46: ^{451}GLGIHSLGLRSLRELGSGLA^{470}
P47: ^{461}SLRELGSGLALIHRNAHLCF^{480}
P48: ^{471}LIHRNAHLCFVHTVPWDQLF^{490}
P49: ^{481}VHTVPWDQLFRNPHQALLHS^{500}
P50: ^{491}RNPHQALLHSGNRPEEDCGL^{510}
P51: ^{501}GNRPEEDCGLEGLVCNSLCA^{520}
P52: ^{511}EGLVCNSLCAHGHCWGPGPT^{530}
P53: ^{521}HGHCWGPGPTQCVNCSHFLR^{540}
P54: ^{531}QCVNCSHFLRGQECVEECRV^{550}
P55: ^{541}GQECVEECRVWKGLPREYVS^{560}
P56: ^{551}WKGLPREYVSDKRCLPCHPE^{570}
P57: ^{561}DKRCLPCHPECQPQNSSETC^{58}[illegible]
P58: ^{571}CQPQNSSETCFGSEADQCAA^{5}[illegible]
P59: ^{581}FGSEADQCAACAHYKDSSSC^{60}[illegible]
P60: ^{591}CAHYKDSSSCVARCPSGVKP^{61}[illegible]
P61: ^{601}VARCPSGVKPDLSYMPIWKY^{62}[illegible]
P62: ^{611}DLSYMPIWKYPDEEGICQPC^{63}[illegible]

Supplemental Figure S5. Sequences of all peptides in rat HER2 overlapping peptide library. A peptide library was constructed based on the extracellular domain sequence of rat ErbB2/HER2. It consisted of 62 peptide 20-mers overlapping by 10 amino acids each Underlined peptides (P9, p17, p35 and p48) were the four focused on in main studies.
